# RNA-Mediated Gene Silencing Signals Are Not Graft Transmissible from the Rootstock to the Scion in Greenhouse-Grown Apple Plants *Malus* sp

**DOI:** 10.3390/ijms13089992

**Published:** 2012-08-10

**Authors:** Henryk Flachowsky, Conny Tränkner, Iris Szankowski, Sascha Waidmann, Magda-Viola Hanke, Dieter Treutter, Thilo C. Fischer

**Affiliations:** 1 Julius Kühn-Institute-Federal Research Centre for Cultivated Plants, Institute for Breeding Research on Horticultural and Fruit Crops, Dresden 01326, Germany; E-Mails: henryk.flachowsky@jki.bund.de (H.F.); viola.hanke@jki.bund.de (M.-V.H.); 2 Plant Breeding Institute, Christian-Albrechts-University of Kiel, Kiel 24098, Germany; E-Mail: c.traenkner@plantbreeding.uni-kiel.de; 3 Division Surgical Research, Regenerative Medicine Program, University and University Hospital Zurich, Zurich 8001, Switzerland; E-Mail: iris.szankowski@usz.ch; 4 Gregor Mendel Institute for Molecular Plant Biology GmbH, Vienna 2000-2419, Austria; E-Mail: sascha.waidmann@gmi.oeaw.ac.at; 5 Unit of Fruit Science, Department for Plant Sciences, Technical University Munich, Freising 85356, Germany; E-Mail: dieter.treutter@wzw.tum.de; 6 Plant Biochemistry and Physiology, Biocenter Botany, Ludwig-Maximilians-University Munich, Planegg-Martinsried 82152, Germany

**Keywords:** anthocyanidin synthase, apple *Malus* spp., graft transmissible, lignification, RNA silencing

## Abstract

RNA silencing describes the sequence specific degradation of RNA targets. Silencing is a non-cell autonomous event that is graft transmissible in different plant species. The present study is the first report on systemic acquired dsRNA-mediated gene silencing of transgenic and endogenous gene sequences in a woody plant like apple. Transgenic apple plants overexpressing a hairpin gene construct of the *gusA* reporter gene were produced. These plants were used as rootstocks and grafted with scions of the *gusA* overexpressing transgenic apple clone T355. After grafting, we observed a reduction of the *gusA* gene expression in T355 scions *in vitro*, but not in T355 scions grown in the greenhouse. Similar results were obtained after silencing of the endogenous *Mdans* gene in apple that is responsible for anthocyanin biosynthesis. Subsequently, we performed grafting experiments with *Mdans* silenced rootstocks and red leaf scions of TNR31-35 in order to evaluate graft transmitted silencing of the endogenous *Mdans*. The results obtained suggested a graft transmission of silencing signals in *in vitro* shoots. In contrast, no graft transmission of dsRNA-mediated gene silencing signals was detectable in greenhouse-grown plants and in plants grown in an insect protection tent.

## 1. Introduction

Gene silencing is an idiom that combines transcription inhibition and RNA degradation. Transcription inhibition is called transcriptional gene silencing (TGS), whereas RNA degradation is called post-transcriptional gene silencing (PTGS) or “RNA interference” [1]. RNA silencing controls development, maintains chromatin, and defends many eukaryotic organisms against viruses [2]. It is based on the existence of partially or perfectly double stranded RNAs (dsRNAs), which are recognized by an RNAse III-like nuclease called DICER-like (DCL) and then become processed into small RNAs (sRNA). At least four different types of silencing pathways that involve different types of sRNAs seem to exist in plants [3]. Based on their origin and biosynthesis, these sRNAs are categorized as micro-RNA (miRNA), trans-acting short interfering (si) RNA (tasiRNA), heterochromatin-associated siRNA (hc-siRNA), and viral siRNA (reviewed in [3]).

In plants sRNAs are incorporated into the RNA induced silencing complex (RISC) containing ARGONAUTE-like (AGO) proteins. After strand separation, the single stranded RNA guides the RISC complex to homologous sequences resulting in transcriptional regulation via DNA/histone methylation or post-transcriptional regulation via mRNA cleavage/destabilization, or translational inhibition of the target sequence (reviewed in [2,3]). In addition, targeted mRNAs can be converted by RNA dependent RNA-polymerases (RDRs) into dsRNAs, which are then processed by DCL to secondary sRNAs [4].

RNA silencing is a non-cell autonomous, mobile process. This is especially the case for siRNAs (e.g., tasiRNAs), which are produced by DCL4 in contrast to many miRNAs, which are mostly produced by DCL1 and not enabled to act non-cell autonomously [5]. After local induction, the silencing effect can spread to adjacent cells or over the whole organism. It can spread from cell to cell over short distances (less than 15 cells), extensive locally (more than 15 cells) or systemically via phloem (reviewed in [1]). The cell to cell transport occurs through plasmodesmata. For spreading over distances exceeding the 15 cell limit, the RNA silencing signal and therewith the silencing effect is amplified by RDRs [6–8]. In contrast to short distance and extensive local silencing, systemic RNA silencing affects the whole plant. Systemic RNA silencing is mediated through signals likely to be transported within the phloem sap. Thereby, the transport through the phloem occurs strictly from source to sink [9]. Systemic silencing can spread from rootstocks to scion [10–12] and vice versa [9,13]. It can pass tissues without complementary sequences and signal amplification [10]. Once the signal reaches the destination (sink) tissue, it is taken from the phloem, amplified and finally transported symplastically between adjacent cells, resulting in gene silencing [6,8].

Graft-transmission of RNA silencing could become of practical importance in horticulture, especially for fruit crops (e.g., apple, pear, grape, and sweet cherry), which are propagated vegetatively by grafting scions of superior clones/cultivars onto clonally propagated rootstocks [14]. The idea to graft non-transgenic scions onto silencing transmitter rootstocks that affect traits within non-transgenic parts of the tree like fruits, seems promising. Beside the advantage of being a straightforward approach to improve individual traits of well-established cultivars such as self-fertility, resistance, flavor, or sugar content, the use of graft-transmissible gene silencing would avoid the possibility of spreading transgenes by outcrossing through pollen and/or seeds. The graft-transmissible manipulation of specific traits provoked by sRNAs has not been demonstrated in apple until now, but it was several times reported in other horticultural plants. For example, wild-type potatoes grafted as stocks with scions overexpressing miR172 showed induced tuberization [15].

Two different approaches allowing a sensitive and visual evaluation of the systemic spread of silencing were developed to prove the possibility of graft-transmitted gene silencing in apple. The first approach is based on silencing of a transgenic reporter gene encoding a β-glucuronidase of *Escherichia coli*. Transgenic apple plants expressing a hairpin (hrp) gene construct of the *gusA* reporter gene were produced, used as rootstocks, and grafted with scions of the transgenic *gusA* overexpressing apple clone T355 [16]. The second approach is based on silencing of an endogenous gene encoding *Malus domestica* anthocyanidin synthase (*Mdans*), which catalyzes the penultimate step in anthocyanin biosynthesis. Transgenic plants expressing a hairpin gene construct of the *Mdans* gene (hrp-*Mdans*) were produced, used as rootstocks, and grafted with the red leaf genotype TNR31-35 of *Malus sieversii* var. *sieversii* f. *Niedzwetzkyana* [17]. Transgenic plants expressing the hrp-*gusA* or the hrp-*Mdans* gene construct were used for grafting experiments *in vitro* and in the greenhouse.

Depending on the gene to be silenced the grafted plants were evaluated by reverse transcription quantitative PCR (RT-qPCR), visually for anthocyanidin coloration, and by histochemical GUS staining, to determine the degree of silencing of the *gusA* transgene and the endogenous *Mdans*, respectively.

## 2. Results

### 2.1. Generation of Hrp-Gusa Transgenic and Hrp-Mdans Transgenic Apple Clones

**Figure 1:**
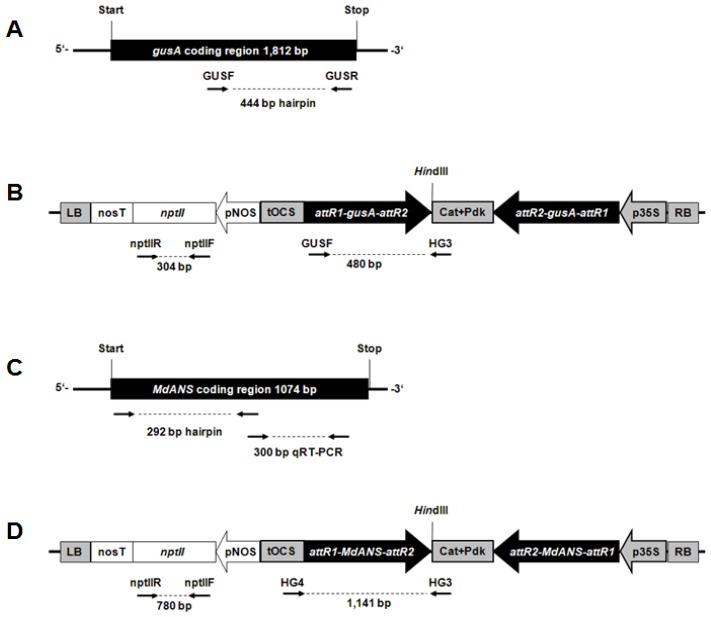
Schematic application of the T-DNAs of the vectors used for plant transformation. (**A**) coding region of the *gusA* gene including the region used for silencing vector construction; (**B**) T-DNA of the *gusA* silencing vector; (**C**) coding region of the *Mdans* gene including the region used for silencing vector construction; (**D**) T-DNA of the *Mdans* silencing vector. Black arrows indicate the position of primers.

A total of three transformation experiments were performed to transform the apple genotype “PinS” with the binary plasmid vector pHELLSGATE8::hrp-*gus* (Figure 1). In total 19 independent putative transgenic plants were obtained after *Agrobacterium tumefaciens*-mediated gene transfer. Thirteen out of them were successfully propagated to establish transgenic clones. Genomic DNA of these clones was isolated from young leaves, in order to examine the integration of the hrp-*gusA* gene construct. For all clones DNA fragments of hrp-*gusA* and *npt*II were amplified by PCR using the primers nptIIF/R for *npt*II and GUSF/HG3 for hrp-*gusA* (Figure S1). Total RNA of each clone was isolated and reverse transcribed into cDNA to determine transgene transcription. RT-PCR analysis using the primers *npt*IIF/R and GUSF/HG3 showed that all clones transcribed both the *npt*II gene and the hrp-*gusA* gene construct (Figure S1). Southern blot analyses were performed using a labeled probe specific for the hrp-*gusA* gene construct. In all transgenic clones hybridization signals were detected indicating the integration of the gene construct.

In total, 26 putative transgenic apple plants were obtained after transformation of “PinS” using *Agrobacterium tumefaciens* strain EHA105 containing the binary plasmid vector pHELLSGATE8::hrp-*Mdans* (Figure 1). These clones were subsequently tested by PCR, Southern blot and RT-PCR on the presence, the integration and expression of the transferred genes. Only seven of these (T1295, T1296, T1297, T1302, T1303, T1308, and T1379) showed consistent results for transgene integration and expression. An example of this investigation is shown in Figure S2. The remaining clones showed no transgene integration except of clone T1378. A fragment of the transferred hrp-*Mdans* gene construct could be amplified by PCR for this clone, but integration of *npt*II was not supported by Southern hybridization. This clone seems to have an imperfect T-DNA integration or a chimeric character. All clones which did not show consistent results in all tests were excluded from further experiments. Clone T1300 which was obviously not transformed was used as additional non-transgenic control.

**Figure 2:**
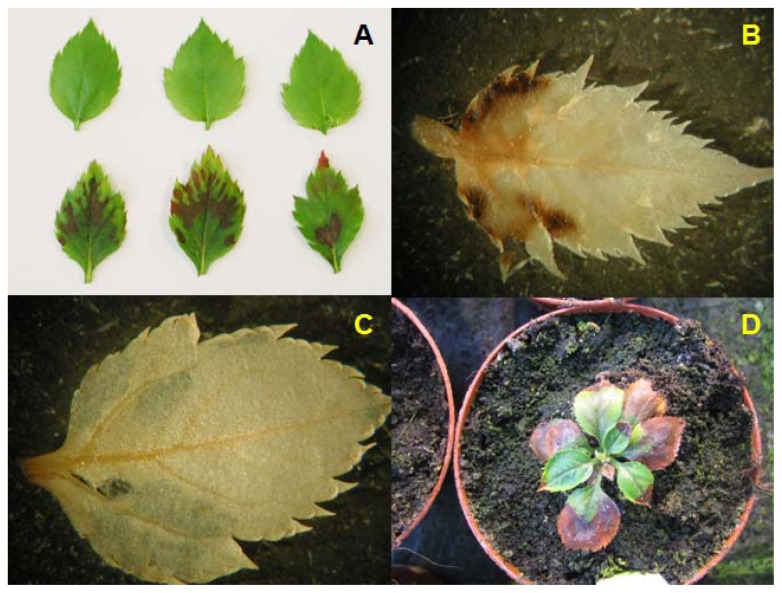
Stress symptoms on leaves of *hrp-Mdans* transgenic apple clones. (**A**) *in vitro* leaves of the non-transformed “PinS” (upper row) without and clone T1302 (lower row) with necrosis; (**B**) leaves of clone T1302 after DAB staining; (**C**) leaves of the non-transformed “PinS” after DAB staining; (**D**) greenhouse plant of clone T1302 showing strong necrosis.

Most of the hrp-*Mdans* transgenic clones died within the next eight months. *In vitro* leaves of these clones went brown (Figure 2A) and the shoots were unable to grow. DAB (3,3-diaminobenzidine) staining suggested a high level of oxidative stress on *in vitro* leaves of the hrp-*Mdans* transgenic clones (Figure 2(B,C)). After transfer to the greenhouse, plants of these clones became necrotic (Figure 2D). Only the two transgenic clones T1297 and T1308, the non-transformed “PinS”, and the non-transgenic clone T1300 were successfully established in the greenhouse.

### 2.2. *In Vitro* Grafting Experiments

**Figure 3:**
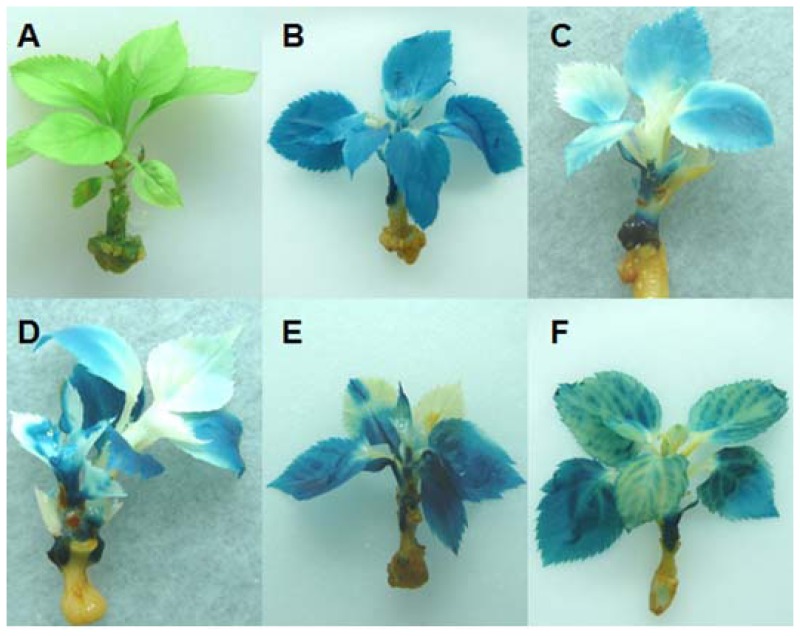

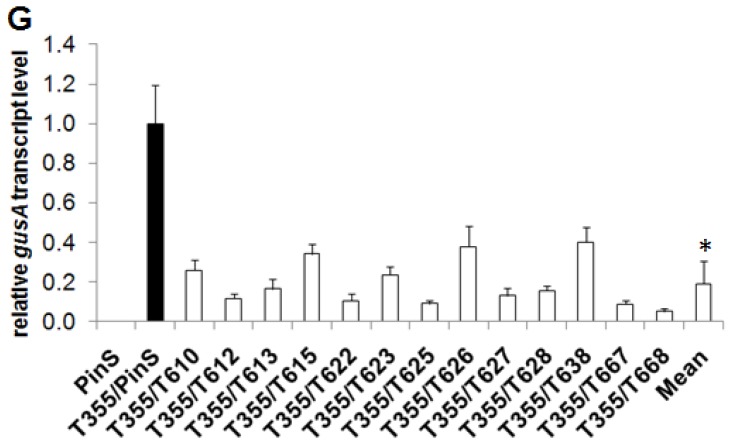
Micrografting experiments with *gusA* transgenic scions of T355 grafted onto non-transformed “PinS” (**A** and **B**) and hrp-*gusA* transgenic rootstocks of clones T612 (**C**), T613 (**D**), T623 (**E**), and T627 (**F**). (**A**) Graftings of T355 onto “PinS” before histochemical β*-glucuronidase* (GUS) staining; (**B**–**F**) Graftings of different genotypes after histochemical GUS staining; (**G**) Detection of the relative *gusA* transcript level in T355 grafted onto “PinS” and hrp-*gusA* transgenic shoots used as rootstocks. The *gusA* transcript levels were determined by Reverse Transcription Quantitative PCR (RT-qPCR). Young leaves of three individual plants were pooled and used for RNA extraction representing one biological replicate. The values are expressed in comparison to T355 grafted onto “PinS”, which was set to be one. The values of T355 grafted onto “PinS” are the mean of three biological replicates of which each was measured in three technical replicates. The values of T355 grafted onto the silencing transmitter clones T610, T612, T613, T615, T622, T623, T625, T626, T628 and T638 are the mean of one biological replicate per clone each measured with three technical replications. The values of T355 grafted onto the silencing transmitter clones T627, T667 and T668 are the mean of two biological replicates each measured with three technical replications. Bars represent standard errors. Mean, mean of all transgenic silencing transmitter clones (T610–T668); * significantly different compared to T355/PinS at α ≤ 0.05.

Micrografting experiments were performed using *in vitro* shoots of the hrp-*gusA* transgenic clones as rootstocks and shoots of the CaMV35S::*gusA* transgenic clone T355 as scions. Furthermore, scions of T355 were also grafted onto non-transgenic “PinS” used as a control (Figure 3A). A total of three to 12 grafted plants per rootstock genotype were established. At the time when the T355 scions had developed five to seven new leaves, young leaf material was collected. Total RNA of these leaves was extracted, reversely transcribed and tested on the level of *gusA* gene transcripts by RT-qPCR. The transcript level of *gusA* was reduced in all *in vitro* micrograftings grown on hrp-*gusA* transgenic rootstocks compared to those grown onto non-transgenic “PinS” (Figure 3G).

**Figure 4:**
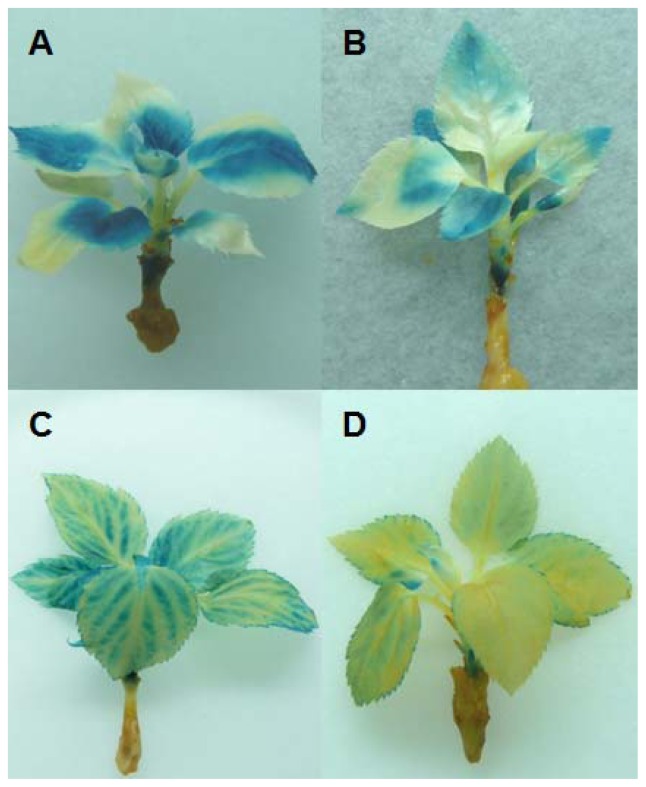
Different types of silencing detected on *gusA* transgenic scions of clone T355 grafted onto hrp-*gusA* transgenic silencing transmitter rootstocks of clones T625 (**A** and **B**) and T668 (**C** and **D**). Shoots grafted onto rootstocks of clone T625 showed locally restricted silencing (type 1). Shoots grafted onto rootstocks of clone T668 showed systemic silencing (type 2).

Four to five weeks after grafting, the grafted plants were evaluated on *gusA* gene expression by histochemical GUS staining. Leaves showing sclerosis or necrosis were excluded from the experiment to ensure that non-blue colored leaf areas are based on silencing. Grafted control plants showed a blue colored scion (T355) and a white rootstock (“PinS”) as expected. Leaves of the T355 scions grafted onto non-transformed “PinS” were nearly blue colored (Figure 3B), resulting the percentage of white (unstained) leaf areas was 7.4 ± 2.2% (mean of 27 individual leaves). In contrast, T355 scions grafted onto hrp-*gusA* transgenic rootstocks were only partial blue colored (Figure 3C–F). Between 19.4 ± 1.3% (clone T610, mean of 15 individual leaves) and 90 ± 12.0% (clone T668, mean of 12 individual leaves) of the entire leaf laminas of the T355 scions were white. The percentage of white colored leaf areas of T355 grafted onto “PinS” and T355 grafted onto the silencing transmitter clones was significantly different at α ≤ 0.001. Silencing of *gusA* affected young and old leaves, spread over entire leaf blades or different parts of the leaves. Two distinct phenotypes of silencing were detected. Type 1 plants showed only parts of leaves that were silenced. Non-silenced parts of these plants/leaves were dark blue colored (Figure 4A,B). The silencing effect was not restricted to a specific leaf area. In type 2 plants, the silencing effect entered the veins and expanded from the veins throughout the whole leaf lamina (Figure 4C,D). Shoots grafted onto the same silencing transmitter genotype (e.g., clone T668) showed always the same phenotype of silencing. The results were reproducible in independent grafting experiments, comprising of two to four grafted plants per experiment.

**Figure 5:**
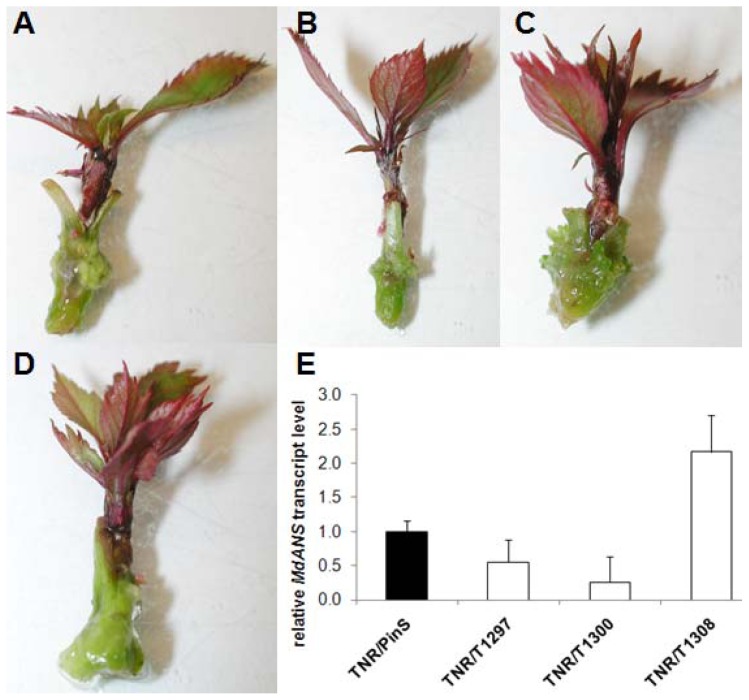
Micrografting experiments with non-transgenic scions of the red leaf *Malus* genotype TNR31-35 grafted onto non-transformed “PinS” (**A**), the hrp-*Mdans* transgenic clones T1297 and T1308 (**B** and **D**), as well as the non-transgenic clone T1300 (**C**). Relative quantification of the *Mdans* mRNA transcript levels in TNR31-35 grafted onto hrp-*Mdans* transgenic apple clones used as rootstocks (**E**). The *Mdans* transcript levels were determined by quantitative real-time PCR (mean of two biological replicates, each three technical repetitions, bars represent standard errors).

*In vitro* shoots of the natural red leaf genotype TNR31-35 were used as scion and grafted onto shoots of the hrp-*Mdans* transgenic clones T1297 and T1308 used as rootstocks. In parallel, shoots of TNR31-35 were also grafted onto non-transgenic “PinS” and clone T1300 used as controls. A total of 30 micrografted plants per genotype were established. After three to four weeks, young leaves of the grafted TNR31-35 were evaluated for leaf coloration or the presence of other visible silencing effects. Surprisingly, no phenotypic changes were detectable. Leaves of TNR31-35 grown on hrp-*Mdans* transgenic clones were normally red colored and could not be distinguished by visible means from those grown on “PinS” (Figure 5(A–D)). Subsequently, young leaves of the grafted TNR31-35 were collected for total RNA extraction. The relative *Mdans* transcript level was determined by RT-qPCR using the primers MdANS_MB1/MB2. The *Mdans* transcript level seemed not to be reduced in leaves of the TNR31-35 shoots grown on hrp-*Mdans* transgenic rootstocks compared to those grown on non-transgenic “PinS” and T1300 (Figure 5E).

### 2.3. Grafting Experiments on Greenhouse Plants

**Figure 6:**
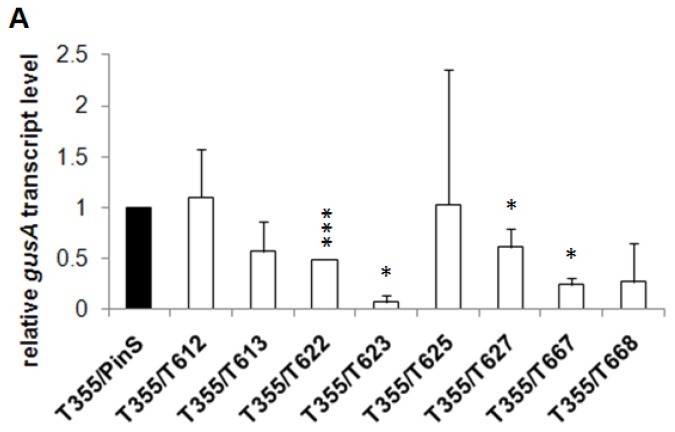

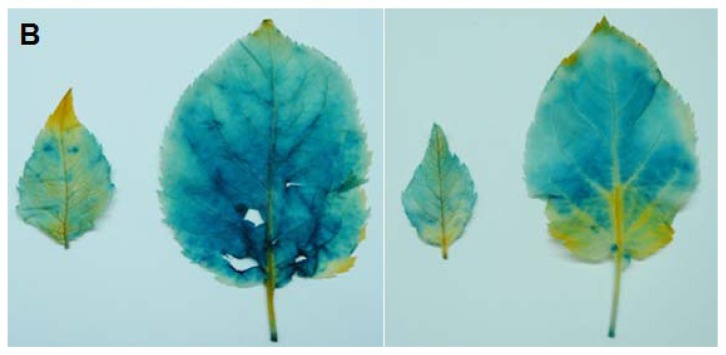
Grafting experiments with greenhouse-grown *gusA* transgenic scions of T355 grafted onto non-transformed “PinS” and hrp-*gusA* transgenic rootstocks of clones T612, T613, T622, T623, T625, T627, T667 and T668. (**A**) Relative quantification of *gusA* mRNA transcript levels in T355 scions grafted onto non-transgenic and hrp-*gusA* transgenic rootstocks, respectively (values mean of three biological replicates, each three technical repetitions, bars represent standard errors). * significantly different to T355/PinS at α ≤ 0.05; *** significantly different to T355/PinS at α ≤ 0.001; (**B**) Histochemical GUS staining of leaves of T335 grafted onto non-transformed “PinS” (**left**) and silencing clone T627 (**right**).

*In vitro* shoots of hrp-*gusA* transgenic clones and non-transformed “PinS” were rooted and transferred to the greenhouse. After one year of cultivation all plants were pruned and grafted with scions of the *gusA* transgenic clone T355. Leaves of each grafted shoot were collected several times within the next two years and tested on *gusA* gene expression by RT-qPCR, histochemically and by colorimetric GUS assay. An example of this is shown in Figure 6. The *gusA* mRNA transcript level often seemed to be reduced (Figure 6A), but this was not supported by the results of histochemical staining (Figure 6B) and colorimetric GUS assay (data not presented). We never found silencing effects that were comparable to those obtained on *in vitro* plants (see Figures 3 and 4).

**Figure 7:**
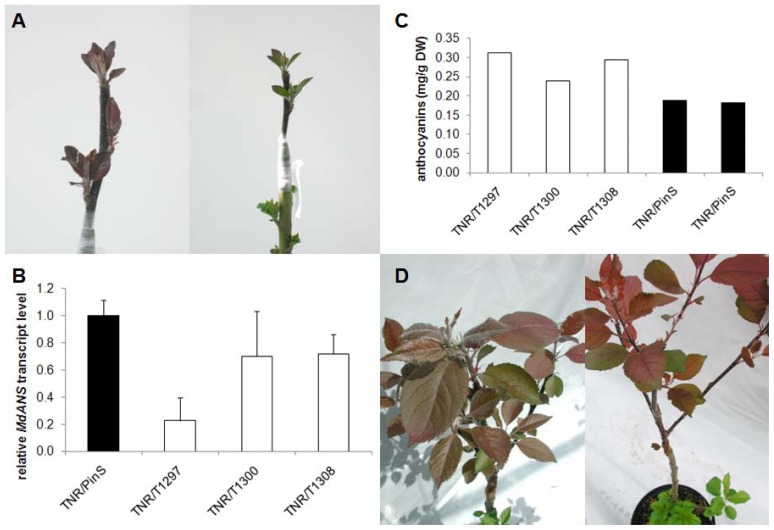
Grafting experiments in the greenhouse with non-transgenic scions of red-leave TNR31-35 grafted onto non-transformed “PinS” and hrp-*Mdans* transgenic rootstocks of clones T1297 and T1308. The non-transformed clone T1300 and “PinS” were used as controls. (**A**) TNR31-35 scions four to five weeks after grafting onto “PinS” (**left**) and T1297 (**right**); (**B**) relative quantification of the *Mdans* mRNA transcript levels in TNR31-35 grafted onto non-transgenic and hrp-*Mdans* transgenic rootstocks, respectively. The *Mdans* transcript levels were determined by quantitative real-time PCR (values mean of three biological replicates, each three technical repetitions, bars represent standard errors); (**C**) *Mdans* silencing dependent changes in anthocyanin contents of TNR31-35 scions grafted onto non-transformed “PinS”, T1300 and hrp-*Mdans* transgenic clones; (**D**) TNR31-35 grafted shoots after one year of greenhouse cultivation. **Left**: TNR31-35 grafted onto “PinS”; **Right**: TNR31-35 grafted onto T1297.

Plants of hrp-*Mdans* transgenic clones and the non-transformed “PinS” were rooted as described and transferred to the greenhouse. After one year of greenhouse cultivation these plants were pruned and used as rootstocks. The rootstocks were grafted with scions of TNR31-35 in order to evaluate the graft transmissible silencing effect of endogenous genes in woody plants. The first leaves, which became visible four to five weeks after grafting, were evaluated on the presence of silencing effects. Leaves of TNR31-35 grown on hrp-*Mdans* transgenic clones appeared to be less intensive red colored then leaves of TNR31-35 grown on “PinS” (Figure 7A). The results obtained by RT-qPCR were not consistent. TNR31-35 shoots grown on the silencing clone T1297 showed a strong reduction of the *Mdans* transcript level, whereas the transcript level was slightly increased in shoots grown on the control T1300 (Figure 7B). The anthocyanin coloration of TNR31-35 shoots grown on hrp-*Mdans* transgenic rootstocks was comparable to that grown on “PinS”. No reduction of the anthocyanin content was detectable (Figure 7C). No differences in leaf coloration were detectable on adult leaves (Figure 7D).

## 3. Discussion

Systemically induced gene silencing from genetically modified (gm) rootstocks to non-gm scions and its application to practical fruit breeding would offer a straightforward approach with significant impact on fruit production and horticulture. Grafting of scions onto silencing transmitter rootstocks for improving individual traits (e.g., self-fertility, resistance or flavor) of cultivars, which are well established in fruit production and on the market place would open a new door for the horticultural practice. The existence of graft-transmissibility of silencing inducing signals has been demonstrated several times in different plant species [9–12,18–21]. Long-distance transport between grafted partners was demonstrated for transcriptional [21] and post-transcriptional gene silencing [22], for transgenes [9,10,20], and for endogenous gene sequences as well [22]. The silencing inducing signal, which moves systemically, is still unknown. There are a number of indications which argue for sRNAs and/or sRNA precursors [3]. In apple *Malus* × *domestica*, where gene silencing has been used effectively several times [23–25], there is no evidence for its systemic spread until today.

Graft-transmission of post-transcriptional gene silencing in apple was tested in the present study using two different approaches. In the first approach, the β-glucuronidase encoding *gusA* gene of *Escherichia coli* was effectively silenced in grafted scions of the transgenic *gusA* overexpressing apple clone T355 of *in vitro* plants. The observed different types of silencing patterns (either complete or partially along vascular tissues, but not gradually in cells in both cases) are evidence for the existence of the whole silencing machinery in apple, which is necessary for its long-distance transport. Furthermore, they may indicate the existence of a critical threshold of siRNAs in cells of grafted scions to switch towards gene silencing. In contrast, for later stages of growth after transfer to greenhouse conditions graft-transmission of silencing was not effective.

In the second approach, the endogenous *Mdans* gene was silenced in transgenic plants of genotype “PinS” used as rootstocks. Several transgenic clones of the apple genotype “PinS” overexpressing a hairpin gene construct of the *MdANS* gene showed a sub-lethal phenotype. This is surprising as this genotype does neither produce anthocyanins in leaves nor in wooden tissue. With vital lines, a systemic spread of silencing was not detectable, neither *in vitro* nor in the greenhouse. Similar results were also obtained in other studies (for review see [1]). Endogenes, like the *Mdans* gene in our study, are known to be protected from the amplification abilities of RDRs. The exclusion from the amplification by RDRs is assumed to be rather correlated with the relatively low steady-state level of mRNA of the endogenes compared to transgenes as with the gene-sequence specificity itself. Therefore, spreading of endogene silencing depends on the long distance transport of the original silencing signal produced in the gm rootstock [1].

The lack of systemic spread of the *Mdans* gene silencing on *in vitro* plants may either be due to the relative low level of *Mdans* gene expression in grafted scions of TNR31-35 in which the *Mdans* gene is actually up-regulated by the *MdMYB10* transcription factor [26,27], or by the lack (or low level) of the original silencing inducing signal.

The systemic transport of signals in plants occurs via the vascular system comprising the phloem and the xylem. A xylem transport of silencing signals would possibly explain the lack of graft-transmission of *gusA* gene silencing in woody plants. The differentiated xylem of trees consists of dead instead of living cells. However, the long distance movement of silencing signals is generally from source to sink through a bulk flow process that is characteristic of the phloem (reviewed in [3]). Based on this fact and the fact that the xylem sap is free of RNA [28] it is generally supposed that silencing is rather transported via phloem than via xylem [3].

The transfer of apple plants from *in vitro* to *ex vitro* conditions is accompanied by lignification of rootstock and scions. Lignification may influence active or passive cell-to-cell transport of siRNAs in living cells. In contrast to *in vitro* plants, *ex vitro* plants possess roots and their habit is multi-branched resulting in a shift in the source to sink relationship. The source to sink direction of *in vitro* shoots is exclusively from older to younger leaves and to the shoot apex whereas the roots of *ex vitro* plants can also represent very strong sinks. Such complex physiological changes associated with the *ex vitro* transfer or lignification can currently not be excluded as a cause for the lack of a systemic silencing effect. Investigation in other woody crop plants for systemic silencing effects seems essential to decide upon a putative general influence of lignification. In case that lignification is generally detrimental for systemic silencing, the use of grafting as a tool to transfer silencing effects from rootstocks to non-transgenic scions seems to be excluded. This would limit the range of applications of silencing in horticulture on the one hand. On the other hand, it would open the possibility to down-regulate specific genes in rootstocks (e.g., growth parameters) without influencing scions. The spatial restriction of the genetic modification within a plant consisting of genetically different partners (e.g., rootstock and scion) is an important point in the current biosafety assessment of gm plants. It is intensively discussed in the study “New Plant Breeding Techniques: State-of-the-Art and Prospects for Commercial Development” which was carried out to respond to an initial request from the Directorate General for the Environment (DG ENV) of the European Commission [29].

## 4. Experimental Section

### 4.1. Vector Design

For cloning of the hrp-*gusA* silencing gene construct a 444 bp fragment of the gene β-glucuronidase (*gusA*) was amplified by PCR using the primers gus-attB1 and gus-attB2 (Table 1). For cloning of the hrp-*Mdans* silencing gene construct a 292 bp fragment of the *Mdans* gene of apple was amplified by PCR using the primers ANS_attB1 and ANS_attB2 (Table 1). Both PCR fragments contained the recombination sites attB1 and attB2, respectively. They were separately cloned into the attR1 and attR2 sites of the binary vector pHELLSGATE8. The cloning into pHELLSGATE8 was performed via the intermediate vector pDONR^TM^ 207 (Invitrogen) containing attP sites in a two step process. PCR, *in vitro* BP and LR clonase recombination reactions were carried out according to the manufacturer’s instructions (Invitrogen). The correct sequence and orientation of the introduced fragments were confirmed by sequencing.

### 4.2. Plant Material and Transformation

Leaves of *in vitro* axillary shoots of a descendant of the apple (*Malus* × *domestica* BORKH.) cultivar “Pinova” (“PinS”) were used for plant transformation. Plant transformation was done as described in [30] using the *A. tumefaciens* strain EHA105 containing the plasmid pHELLSGATE8::hrp-*gus* and the plasmid pHELLSGATE8::hrp-*Mdans*, respectively. Cultivation and micropropagation of *in vitro* shoots were also realized as described in [30]. Furthermore, the CaMV35S::*gusA* transgenic apple clone T355 described by Flachowsky *et al*. [16] and the red leaf genotype TNR31-35 (hybrid of *Malus sieversii* var. *sieversii* f. *niedzwetzkyana*, JKI collection Pillnitz, Germany) were used.

### 4.3. Molecular Evaluation of Transgenic Plants

Genomic DNA was extracted from leaf tissue with the DNeasy^®^ Plant Mini Kit (Qiagen). Standard PCR assays were performed in a total volume of 25 μL containing 50 ng DNA, 1 × NH_4_-buffer, 1.5 mM MgCl_2_, 0.2 mM dNTPs, 0.5 μM of each primer and 0.5 U Taq DNA polymerase (Invitrogen). The PCR conditions were 35 cycles of 30 s denaturation at 94 °C, 1 min annealing (temperature depended on the primers used) and 1 min elongation at 72 °C. The length of PCR products was determined by electrophoresis on a 1% agarose gel with a 100 bp molecular size marker (MBI Fermentas). All primers used in this study are listed in Table 1.

For transcription analyses, total RNA was isolated from leaves using the Invisorb^®^ Spin Plant RNA Mini Kit (Invitek), followed by a *DNase*I treatment (Ambion). Presence of DNA contamination was tested by standard PCR using 2 μL RNA as template and the primers EF_F/EF_R, which are specific for the elongation factor gene *EF1α* of apple. The remaining RNA was reverse transcribed using oligo(dT)_18_ primers and the RevertAid™ First Strand cDNA Synthesis Kit (MBI Fermentas). The subsequent PCR reactions were performed with 1 μL cDNA and gene specific primers (Table 1).

Southern hybridization was performed as described by Flachowsky *et al*. [31]. Transgene integration was detected with digoxygenin (DIG)-labeled probes generated by PCR using the primer pairs GusF/HG3 for the hrp-*gusA* gene construct and *npt*IIF/*npt*IIR for the selectable marker gene *npt*II (Table 1).

### 4.4. Reverse Transcription Quantitative PCR (RT-qPCR)

RT-qPCR was performed as described by Flachowsky *et al*. [31]. Amplification and correlation efficiencies of each PCR assay were determined on diluted plasmid DNA of the transformation vectors. The expression of the transgenes as well as for the endogenous gene*s* was studied using the gene specific primers listed in Table 1. All expression data were normalized using apple *β-actin* or *RNA polymerase subunit II* (*RNApolII*) as internal control for each sample.

### 4.5. Grafting Experiments

Micrografting of *in vitro* grown plants was performed as described by Tränkner *et al*. [32]. For grafting experiments in the greenhouse, micropropagated *in vitro* shoots were rooted and acclimatized to greenhouse conditions as described by Flachowsky *et al*. [30]. Grafting of greenhouse plants was performed using the whip-and-tongue grafting technique as described by Crasweller [33].

### 4.6. Histochemical GUS Assay

For GUS staining assays, the plant material was completely covered with X-Gluc solution containing 50 mM sodium phosphate pH 7.0, 1 mM EDTA, 0.2% Triton X 100, 0.05% SDS, 0.033% *N*-Lauryl-Sarcosine, 1 mM potassium ferricyanide and 50 mg X-Gluc (MBI Fermentas). After 1 h vacuum infiltration, the plants were incubated over night at 37 °C. The chlorophyll was removed with ethanol-acetic acid solution in a ratio of 3:1 for 16 h at room temperature. The software program Carnoy 2.0 (Alnini, Inc.: Petaluma, CA, USA, 2012) was used to measure silenced and non-silenced areas after GUS staining.

### 4.7. Anthocyanin Coloration

Anthocyanidin coloration was evaluated visually for this study. Analytical verification of color by apple anthocyanins after overexpression or direct silencing of anthocyanidin synthase in a red-leaved cultivar has been performed as described by Li *et al*. [13] and Szankowski *et al*. [23].

### 4.8. Statistical Analysis

Quantitative data were subjected to statistical analysis (ANOVA and Duncan’s multiple range test) using the SAS^®^ 9.1 software (SAS Institute: Cary, NC, USA, 2004).

## 5. Conclusions

It has been demonstrated for the first time that a silencing signal is transported from a transgenic rootstock of apple (*Malus* × *domestica*) to *in vitro* grafted scions over expressing the heterologous reporter gene β-glucuronidase. This transport of a silencing signal in *in vitro* graftings is not seen when the endogenous anthocyanidin synthase gene is used as a reporter gene. *Ex vitro* under greenhouse conditions the transport of the silencing signal for the heterologous reporter gene β-glucuronidase is also not observed. Hypothetically, this is correlated with the lignification process in tissues under these conditions. For final conclusion, this should be compared with results to be obtained with other woody plant systems.

## Supplementary Materials



## Figures and Tables

**Table 1:** Sequences of the primers used in this study.

Primer		Sequence 5′→3′
*gus*-attB	B1	GGGGACAAGTTTGTACAAAAAAGC
		AGGCTGTTCTGCGACGCTCACACCGATACC
	B2	GGGACCACTTTGTACAAGAAAGCT
		GGGTTCACCGAAGTTCATGCCAGTCCAG
*ANS*_attB	B1	GGGGACAAGTTTGTACAAAAAAGC
		AGGCTCTGTGAGCTCTGATTCAGTGA
	B2	GGGACCACTTTGTACAAGAAAGCT
		GGGTACCTTGTCCATGAGCTCGTCA
*npt*II	F	GGTTCTCCGGCCGCTTGGGTG
	R	CGGCAGGAGCAAGGTGAGATGAC
GUS	F	GTTCTGCGACGCTCACACCGATACC
	R	TCACCGAAGTTCATGCCAGTCCAG
EF	F	ATTGTGGTCATTGG(CT)CA(CT)GT
	R	CCAATCTTGTA(AGC)ACATCCTG
Act	F	GTGAGGCTCTATTCCAACCATC
	R	GGAACACAAATTGGGCAAGTAT
HG	1	GCAAGTGGATTGATGTGACATCTCC
	3	CGTCTGTGATGGCTTCCATGTCGGC
	3n	GGATCCTCTAGACCACTTTGTAC
	4	CGAAACCGGCGGTAAGGATCTGAGC
EF1a	F	ATTGTGGTCATTGGYCAYGT
	R	CCAATCTTGTAVACATCCTG
RNApolII	F	ATATGCCACCCCGTTCTCTACT
	R	CACGTTCCATTTGTCCAAACTT
*MdANS*	MB1	CACCTTCATCCTCCACAACAT
	MB2	ATGTGCTCAGCAAAAGTTCGT
	F	GTGAGCTCTGATTCAGTGA
	R	CCTTGTCCATGAGCTCGTCA

## Abbreviations

*ans*: anthocyanidin synthase
*gus*: β-glucuronidase
