# Supplementary material for: RNA-Mediated Gene Silencing Signals Are Not Graft Transmissible from the Rootstock to the Scion in Greenhouse-Grown Apple Plants Malus sp
Source: Int J Mol Sci. 2012 Aug 10;13(8):9992–10009. doi: 10.3390/ijms13089992 (PMC3431842; doi:10.3390/ijms13089992)
Supplement: Supplementary file 1 [file ijms-13-09992-s001.pdf]

RNA-Mediated Gene Silencing Signals Are Not Graft Transmissible from the Rootstock to the Scion in Greenhouse-Grown Apple Plants *Malus* sp.

Supplementary Information

Figure S1. Molecular evaluation of hrp-*gusA* transgenic apple clones by PCR (**A**) and RT-PCR; (**B**) on genomic DNA and cDNA, respectively. H_2O, blank probe; plasmid, positive control pHELLSGATE::hrp-*gusA* (the vector PCR 2.1 Topo containing a genomic clone of EF1alpha was used as control for the EF1a RT-PCR), PinS, descendent of the cultivar "inova". Transgenic clone T667 was separately tested by RT-PCR (data not presented).

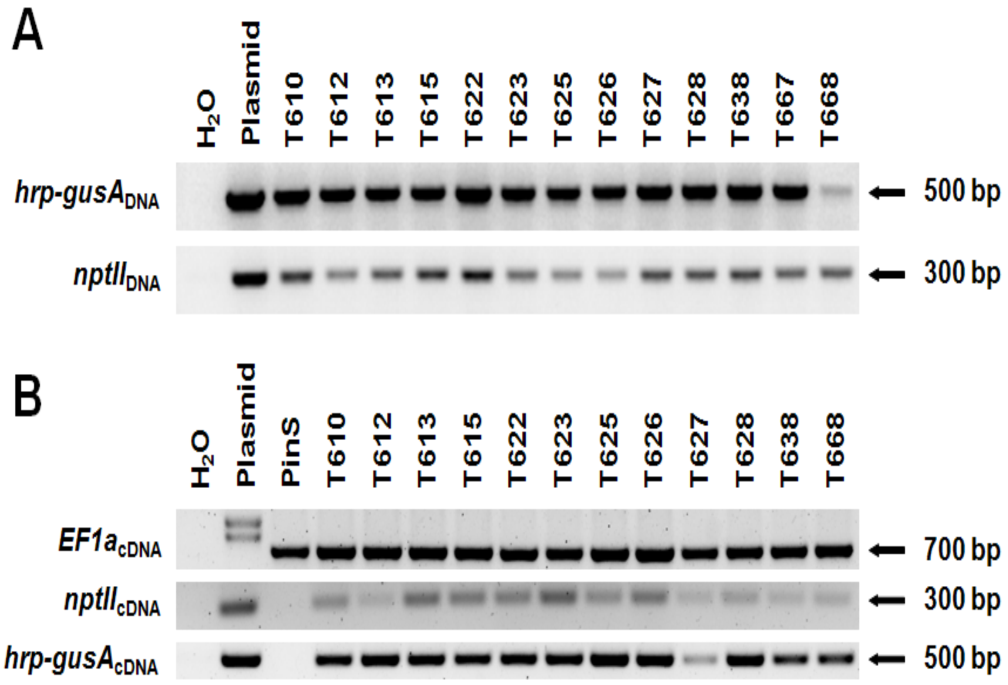

Figure S2. Molecular evaluation of hrp-*Mdans* transgenic plants. (**A**) PCR based detection of the hrp-*Mdans* hairpin gene construct on genomic DNA using the primers HG1 and HG3n; (**B**) Southern blot hybridization of *Sac*I digested DNA using a DIG-labeled probe of the *nptII*; (**C**) Relative mRNA expression of the *Mdans* gene measured on *in vitro* grown plants (mean of three biological replicates, each with three technical replicates); (**D**) Relative mRNA expression of the *Mdans* gene measured on greenhouse grown plants (mean of three biological replicates, each with three technical replicates). SM, molecular size marker (100 bp DNA ladder, MBI Fermentas); MW, 1 kbp, molecular weight marker II, DIG labelled (Roche); "PinS", descendant of the apple cultivar "Pinova".

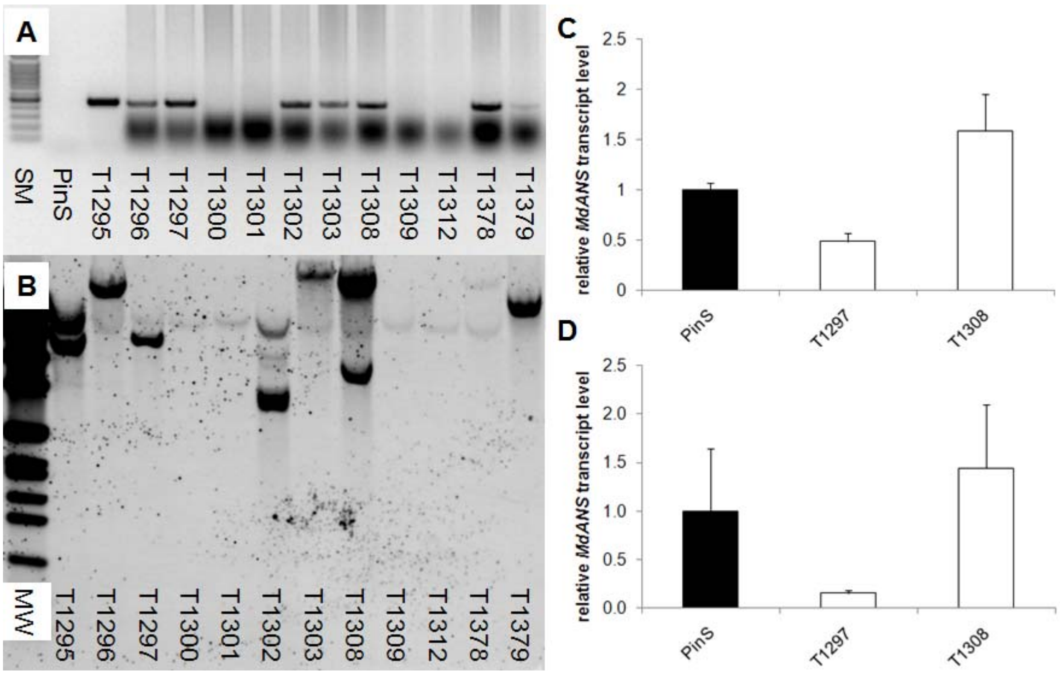

© 2012 by the authors; licensee MDPI, Basel, Switzerland. This article is an open access article distributed under the terms and conditions of the Creative Commons Attribution license (http://creativecommons.org/licenses/by/3.0/).
